# Supplementary material for: Intestinal AMPK modulation of microbiota mediates crosstalk with brown fat to control thermogenesis
Source: Nat Commun. 2022 Mar 3;13:1135. doi: 10.1038/s41467-022-28743-5 (PMC8894485; doi:10.1038/s41467-022-28743-5)
Supplement: Supplementary file 1 — Supplementary Information [file 41467_2022_28743_MOESM1_ESM.pdf]

Supplementary Information

Intestinal AMPK modulation of microbiota mediates crosstalk with brown fat to control thermogenesis

Eryun Zhang et al.

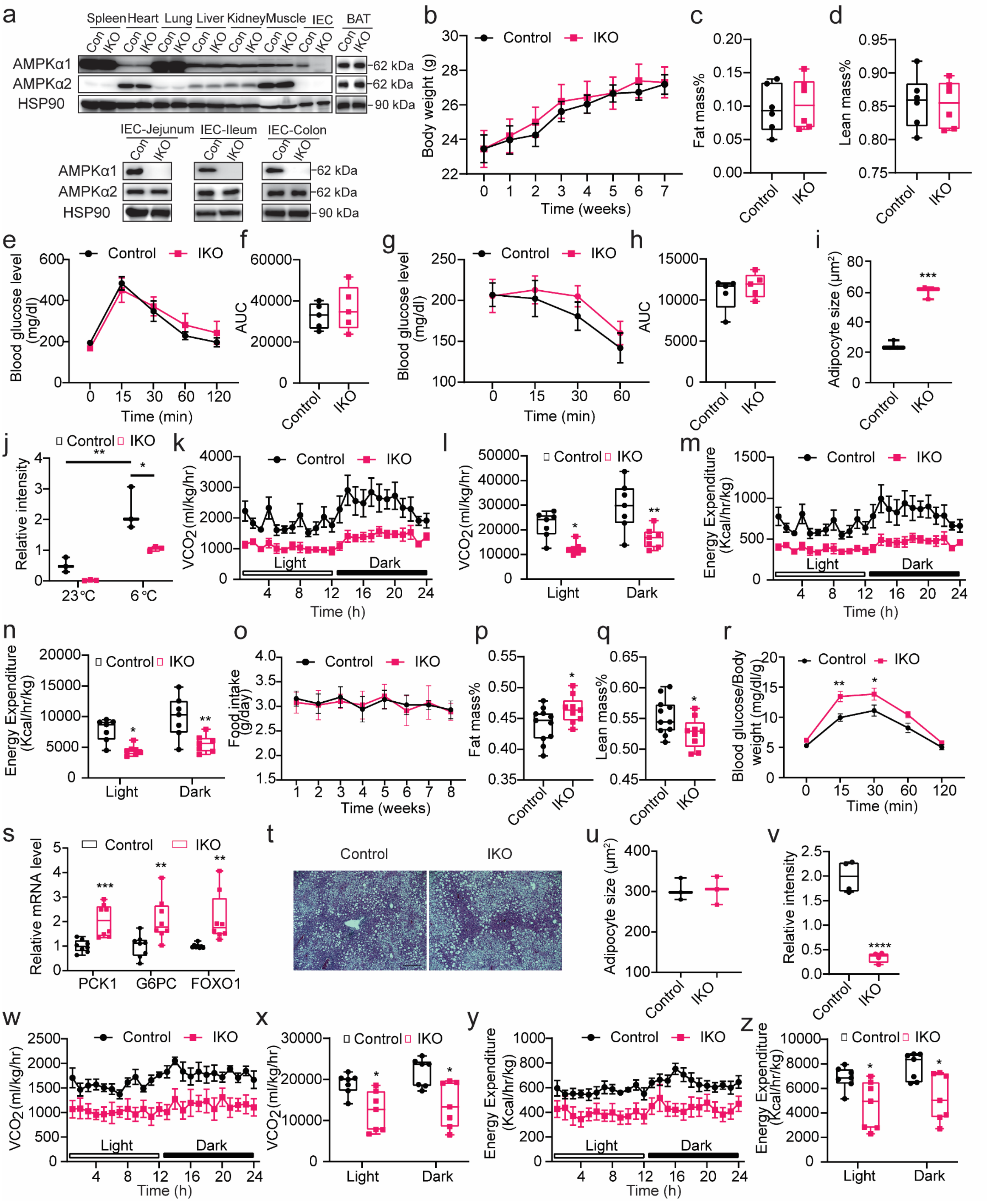
a
Spleen Heart Lung Liver Kidney Muscle IEC BAT
AMPKα1
AMPKα2
HSP90
62 kDa
90 kDa
IEC-Jejunum IEC-Ileum IEC-Colon
Con IKO
b
Control IKO
Body weight (g)
Time (weeks)
c
Fat mass%
d
Lean mass%
e
Blood glucose level (mg/dl)
Time (min)
f
AUC
g
h
i
Adipocyte size (μm²)
j
Relative intensity
23 °C
6 °C
k
VCO2 (ml/kg/hr)
Light
Dark
Time (h)
l
m
Energy Expenditure (Kcal/hr/kg)
n
o
Food intake (g/day)
p
q
r
Blood glucose/Body weight (mg/dl/g)
s
Relative mRNA level
PCK1 G6PC FOXO1
t
Control IKO
u
v
w
x
y
z

Supplementary Fig. 1 Metabolic phenotypes of AMPKα1-IKO mice. For a-n, mice were fed chow diet. (a) Western blot analysis of AMPKα1 protein in various tissues of AMPKα1$^{fl/fl}$ (Con) and AMPKα1-IKO mice.(b) Body weight of mice from 6-week-old (n=7 biologically independent samples for control group and n=6 biologically independent samples for IKO group). (c-d) The ratio of fat mass (c) and lean mass (d) to the body weights of mice (n=6 biologically independent samples). (e-h) Glucose tolerance test (e, f) and insulin tolerance test (g, h) results, including blood glucose levels and the area under the curve (AUC) for 12-week-old mice (n=5 biologically independent samples). (i) Adipocyte size analysis in Figure 1a (n=3 biologically independent samples), *P* value: 0.0003. (j) The quantified densities of the Western blot bands in Figure 1c (n=3 biologically independent samples), *P* value: 0.0023, 0.0210. (k-n) VCO_2 (k-l) and energy expenditure (m-n) of mice over 24 h (n=7 biologically independent samples), *P* value for l: 0.0009, 0.0081. *P* value for n: 0.0010, 0.0085. For o-z, mice were fed HFD. (o) Daily food intake (n=5 biologically independent samples). (p-q) The ratio of fat mass (p) and lean mass (q) to the body weights of mice. (n=11 biologically independent samples for control group and n=9 biologically independent samples for IKO group), *P* value for p:0.0448. *P* value for q:0.0439. (r) Glucose tolerance test relative to the body weight at 10 weeks of HFD feeding (n=7 biologically independent samples for control group and n=5 biologically independent samples for IKO group), *P* value:0.0025, 0.0271. (s) Gene expression of PCK1, G6PC and FOXO1 in the liver tissue (n=8 biologically independent samples), *P* value:0.0008, 0.0074, 0.0066. (t-u) Representative images (t) and lipid droplet size analysis (u, n=3 biologically independent samples) of H&E-stained liver sections. Scale bar =100 μm. (v) The quantified densities of the Western blot bands in Figure 1m (n=7 biologically independent samples). *P* value: <0.0001. (w-z) VCO_2 (w-x) and energy expenditure (y-z) of mice (n=7 biologically independent samples). *P* value for x:0.0071, 0.0078. *P* value for z:0.0189, 0.0105. Values are means ± s.e.m. for b, e, g, k, m, o, r, w, y. The boxplot elements (for c-d, f, h-j, l, n, p-q, s, u-v, x, z) are defined as following: center line, median; box limits, upper and lower quartiles; whiskers, 1.5 × interquartile range. *P<0.05, **P<0.01, ***P<0.001 and ****P<0.0001 cf. Control mice by two-tailed Student's t-tests (for c-d, f, h-i, l, n, p-q, s, u-v, x, z), and two-way ANOVA with Tukey's post-hoc tests (for b, e, g, j, o, r). Source data are provided as a Source Data file.

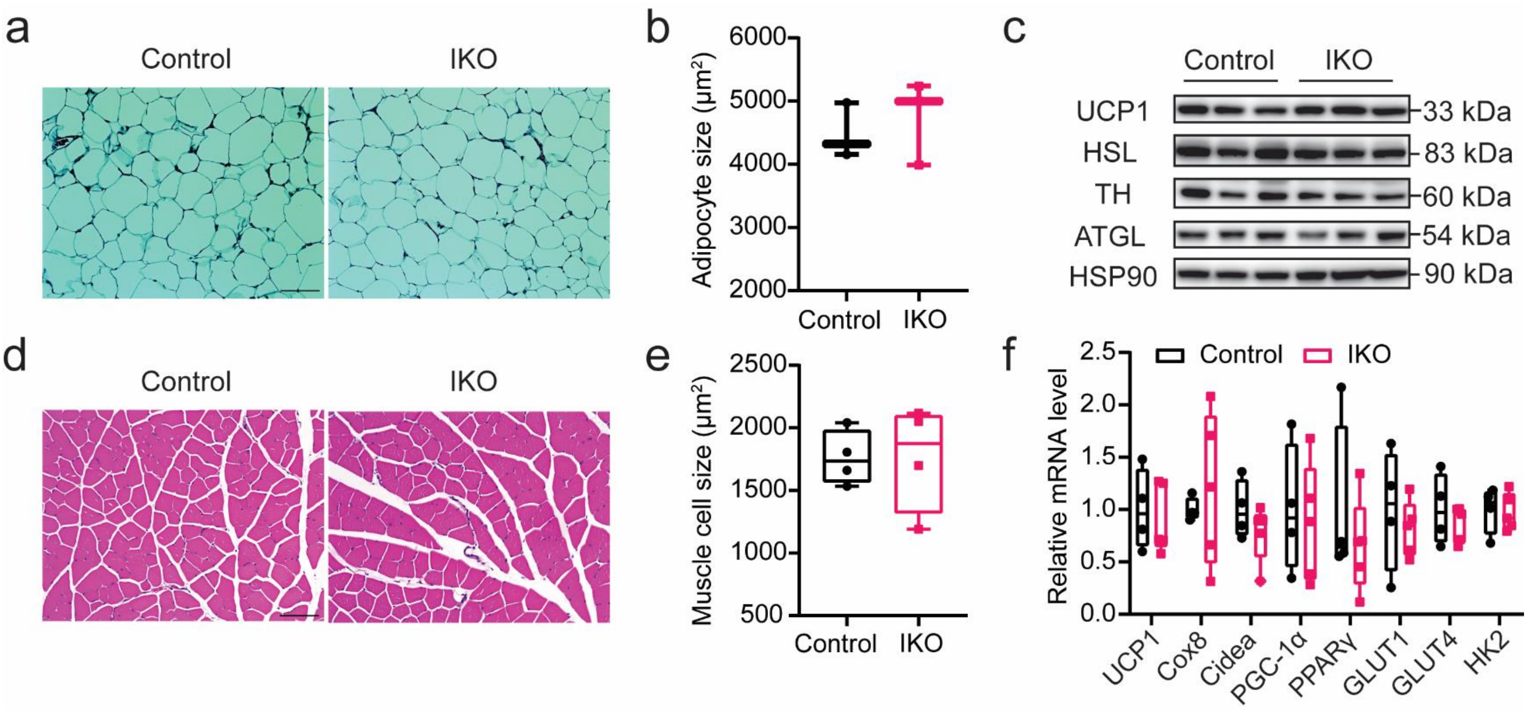

Supplementary Fig. 2. Phenotype of inguinal WAT (iWAT) and muscle in the HFD-fed AMPKα1$^{fl/fl}$ and IKO mice. (a-b) Representative images (a) and adipocyte size analysis (b, n=3 biologically independent samples) of H&E-stained iWAT sections. Scale bar =100 μm. (c) Representative Western blot analysis of UCP1 protein levels in the iWAT. (d-e) Representative images (e) and muscle cell size analysis (f, n=4 biologically independent samples) of H&E-stained muscle sections. Scale bar =100 μm. (f) Relative mRNA levels of genes expressed in the muscle (n=4 biologically independent samples for control group and n=5 biologically independent samples for IKO group). The boxplot elements (for b-c, e-f) are defined as following: center line, median; box limits, upper and lower quartiles; whiskers, 1.5 × interquartile range. Source data are provided as a Source Data file.

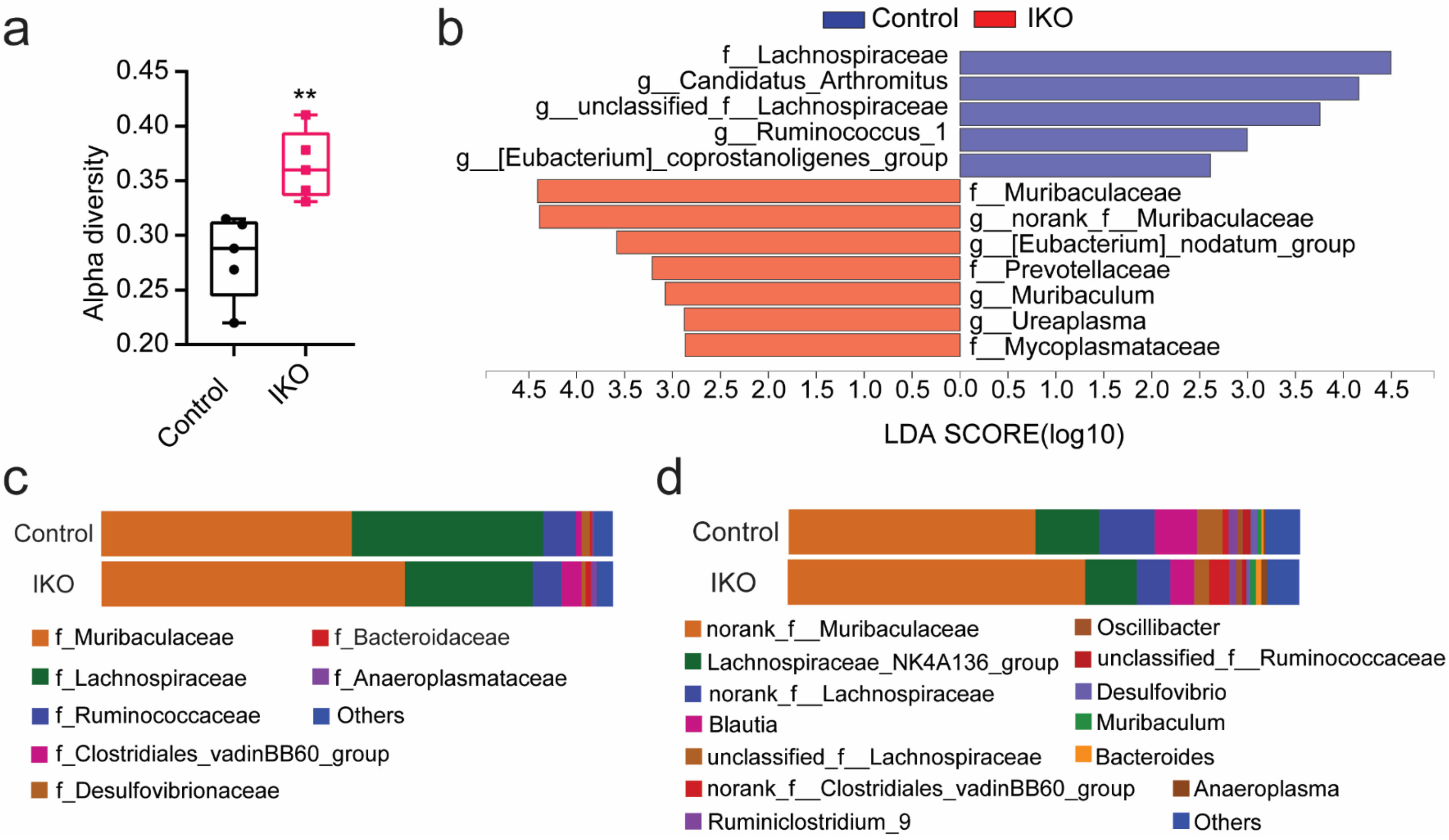

Supplementary Fig. 3 Alteration of gut microbiota profile in AMPKα1-IKO mice. (a) Alpha diversity analysis of the gut microbiota in AMPKα1$^{fl/fl}$ (Control) and AMPKα1-IKO mice fed chow diet (n=5 biologically independent samples), *P* value: 0.0055. (b) Linear discriminant analysis (LDA) effect size (LEfSe) score was calculated to evaluate bacterial families or genera overrepresented in mice fed chow diet (n=5 biologically independent samples). (c, d) Bacterial taxon-based analysis at the family level (c) and genus level (d) in mice fed chow diet (n=5 biologically independent samples). The boxplot elements (for a) are defined as following: center line, median; box limits, upper and lower quartiles; whiskers, 1.5 × interquartile range. ***P* < 0.01 by two-tailed Student's t-test. Source data are provided as a Source Data file.

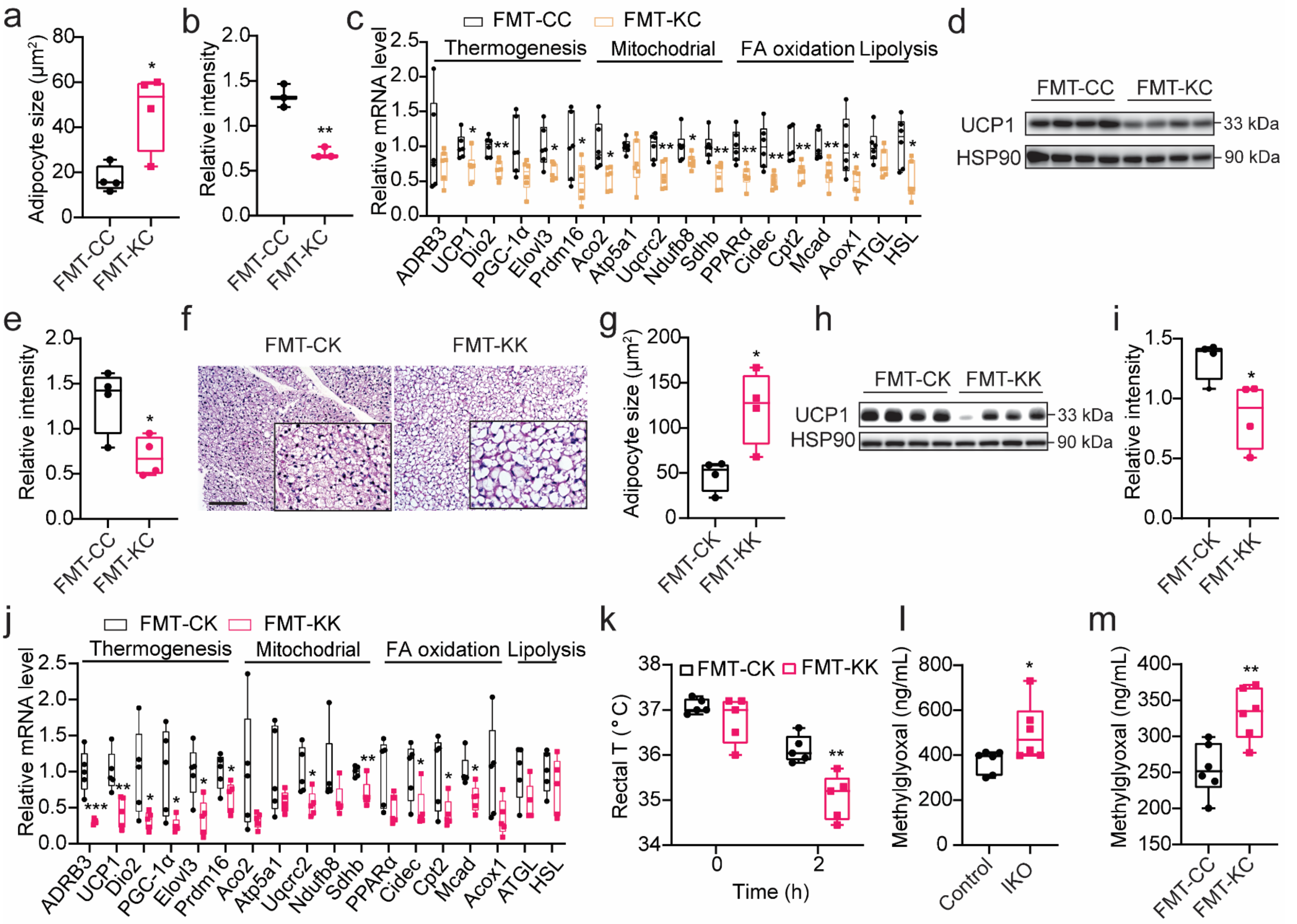

a
Adipocyte size (μm²)
FMT-CC
FMT-KC
b
Relative intensity
FMT-CC
FMT-KC
c
FMT-CC
FMT-KC
Thermogenesis
Mitochodrial
FA oxidation
Lipolysis
Relative mRNA level
ADRB3
UCP1
Dio2
PGC-1α
Elovl3
Prdm16
Aco2
Atp5a1
Uqcrc2
Ndufb8
Sdhb
PPARα
Cidec
Cpt2
Mcad
Acox1
ATGL
HSL
d
FMT-CC
FMT-KC
UCP1
HSP90
33 kDa
90 kDa
e
Relative intensity
FMT-CC
FMT-KC
f
FMT-CK
FMT-KK
g
Adipocyte size (μm²)
FMT-CK
FMT-KK
h
FMT-CK
FMT-KK
UCP1
HSP90
33 kDa
90 kDa
i
Relative intensity
FMT-CK
FMT-KK
j
FMT-CK
FMT-KK
Thermogenesis
Mitochodrial
FA oxidation
Lipolysis
Relative mRNA level
ADRB3
UCP1
Dio2
PGC-1α
Elovl3
Prdm16
Aco2
Atp5a1
Uqcrc2
Ndufb8
Sdhb
PPARα
Cidec
Cpt2
Mcad
Acox1
ATGL
HSL
k
FMT-CK
FMT-KK
Rectal T (° C)
Time (h)
l
Methylglyoxal (ng/mL)
Control
IKO
m
Methylglyoxal (ng/mL)
FMT-CC
FMT-KC

Supplementary Fig. 4 Gut microbiota alteration in AMPKα1-IKO mice are sufficient to impair BAT thermogenesis. Fresh feces from AMPKα1$^{fl/fl}$ and AMPKα1-IKO mice were transplanted to WT or AMPKα1-IKO recipient mice. FMT-CC, FMT from AMPKα1$^{fl/fl}$ mice to WT mice; FMT-KC, FMT from AMPKα1-IKO mice to WT mice. FMT-CK, FMT from AMPKα1$^{fl/fl}$ mice to AMPKα1-IKO mice. FMT-KK, FMT from AMPKα1-IKO mice to AMPKα1-IKO mice. (a) Adipocyte size analysis in Figure 2c (n=4 biologically independent samples), *P* value: 0.0162. (b) The quantified densities of the Western blot bands in Figure 2e (n=3 biologically independent samples), *P* value: 0.0015. (c) Relative mRNA levels of genes expressed in the BAT of recipient mice exposed to cold for 6 h (n=6 biologically independent samples), *P* value: 0.0209, 0.0016, 0.0129, 0.0442, 0.0159, 0.0027, 0.0471, 0.0026, 0.0031, 0.0039, 0.0067, 0.0069, 0.0216, 0.0100. (d-e) Western blot analysis of UCP1 protein levels in the BAT of recipient mice exposed to cold for 6 h (n=4 biologically independent samples), *P* value: 0.0262. (f-g) Representative images (f) and adipocyte size analysis (g, n=4 biologically independent samples) of H&E-stained BAT sections from FMT recipient mice. Scale bar =100 µm. *P* value: 0.0152. (h-i) Western blot analysis of UCP1 protein levels in the BAT of recipient mice (n=4 biologically independent samples), *P* value: 0.0272. (j) Relative mRNA levels of genes expressed in the BAT of recipient mice. (n=6 biologically independent samples), *P* value:0.0007, 0.0069, 0.0388, 0.0273, 0.0105, 0.0380, 0.0235, 0.0084, 0.0445, 0.0480, 0.0200. (k) Rectal temperatures of recipient mice exposed to 6 ºC for 2 h (n=5 biologically independent samples), *P* value: 0.0013. (l) Methylglyoxal levels in the fecal samples in the chow diet-fed AMPK mice measured by LC-MS/MS methods (n=6 biologically independent samples), *P* value: 0.0405. (m) Methylglyoxal levels in the fecal samples in FMT recipient mice measured by LC-MS/MS methods (n=6 biologically independent samples), *P* value: 0.0041. The boxplot elements (for a-c, e, g, i-m) are defined as following: center line, median; box limits, upper and lower quartiles; whiskers, 1.5 × interquartile range. **P* < 0.05, ***P* <0.01 and ****P* <0.01 by two-tailed Student's t-tests (for a-c, e-g, i-j, l), and two-way ANOVA with Tukey's post-hoc tests for m. Source data are provided as a Source Data file.

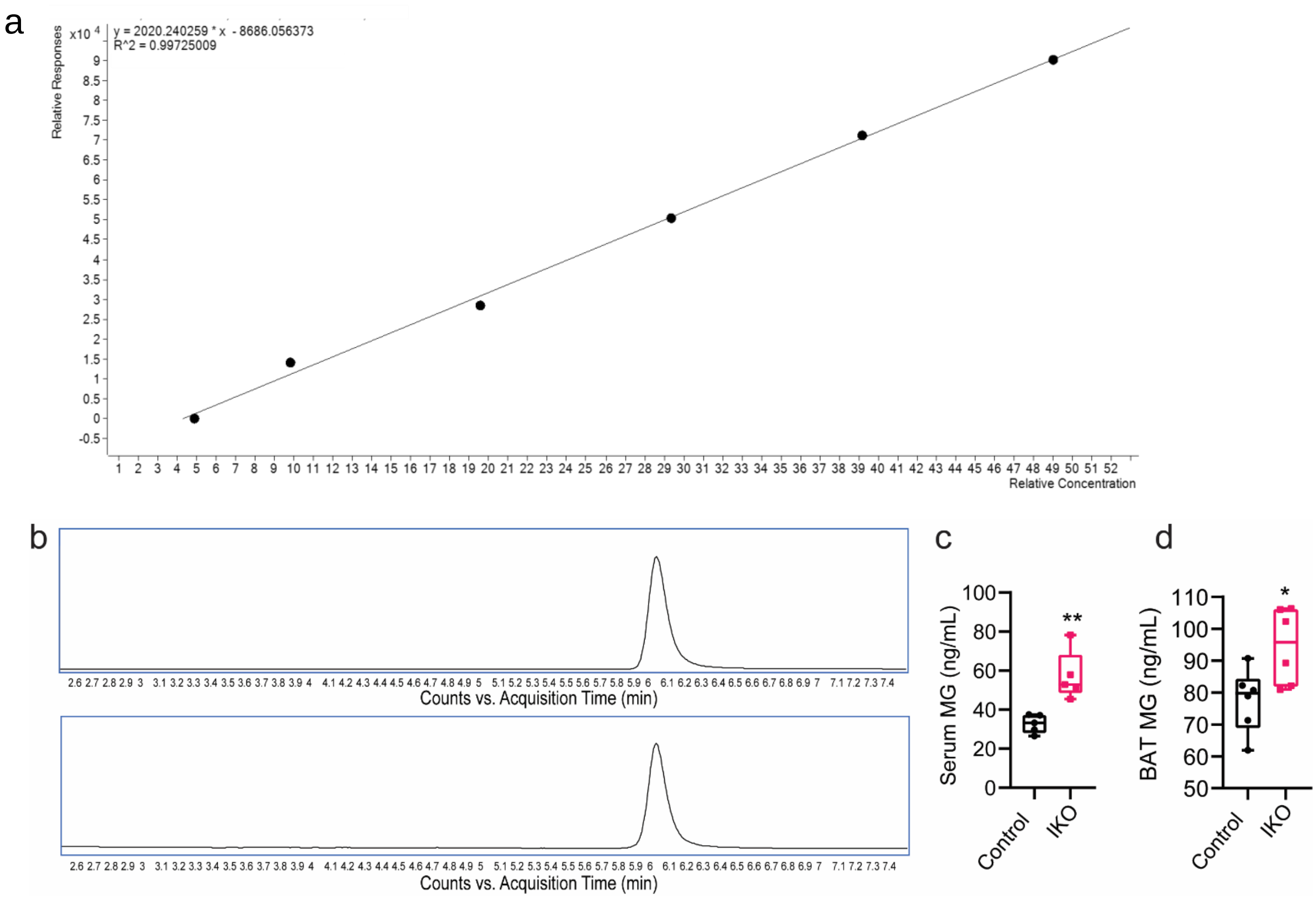

Supplementary Fig. 5 The analysis of methylglyoxal using by LC-MS/MS. (a) The standard curve. (b) the sample curve (below) matched with the standard curve (upper). (c) MG levels in the serum of DIO mice (n=5 biologically independent samples), *P* value: 0.0036. (d) MG levels in the BAT tissues of chow diet-fed mice (n=6 biologically independent samples), *P* value: 0.0230. The boxplot elements (for c-d) are defined as following: center line, median; box limits, upper and lower quartiles; whiskers, 1.5 × interquartile range. **P*<0.05, ***P*<0.01 by two-tailed Student's t-tests. Source data are provided as a Source Data file.

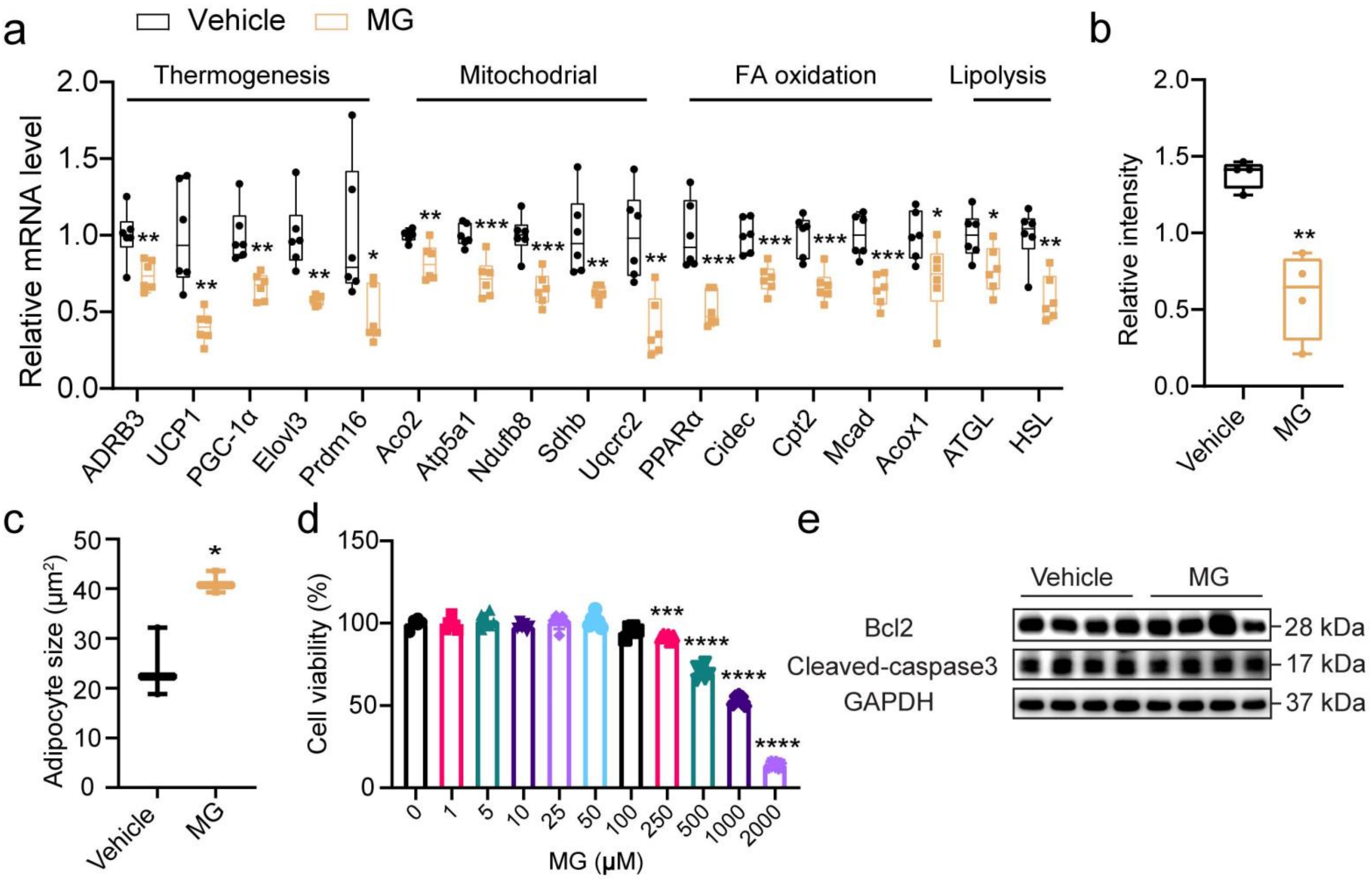

Supplementary Fig. 6 Methylglyoxal treatment reduces the expression of functional genes in brown adipocyte. (a) Gene expression in mature HIB1B cell line treated with 50 μM methylglyoxal (n=6 biologically independent samples), *P* value: 0.0086, 0.0019, 0.0021, 0.0010, 0.0246, 0.0063, 0.0008, 0.0004, 0.0060, 0.0018, 0.0008, 0.0006, 0.0007, 0.0005, 0.0348, 0.0268, 0.0014. (b) The quantified densities of the Western blot bands in Figure 2k (n=4 biologically independent samples), *P* value: 0.0018. (c) Adipocyte size analysis in Figure 2i (n=3 biologically independent samples), *P* value: 0.0163. (d) Cell viability test in the HIB1B cells treated with gradient concentrations of Methylglyoxal (MG) (n=6 biologically independent samples), *P* value: 0.0006, <0.0001, <0.0001, <0.0001. (e) Bcl2 and Cleaved-caspase3 protein expression in the BAT of WT mice after i.p. injections of methylglyoxal (50 mg/kg) once daily for 2 weeks. The boxplot elements (for a-d) are defined as following: center line, median; box limits, upper and lower quartiles; whiskers, 1.5 × interquartile range. *P<0.05, **P<0.01, ***P<0.001 and ****P<0.0001 by two-tailed Student's t-tests or one-way ANOVA with Tukey's post-hoc tests. Source data are provided as a Source Data file.

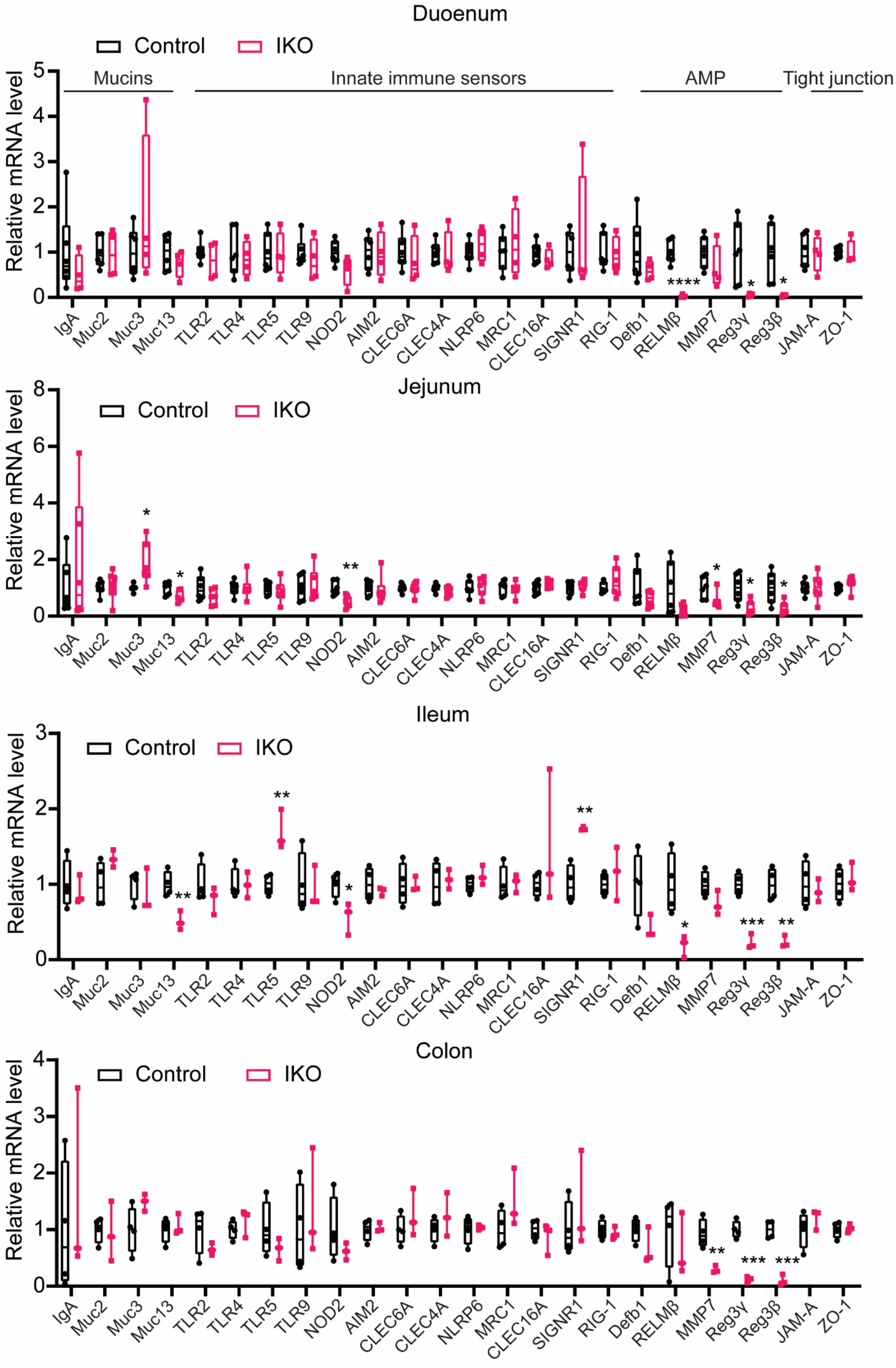

Duoenum
Control
IKO
Relative mRNA level
Mucins
Innate immune sensors
AMP
Tight junction
IgA
Muc2
Muc3
Muc13
TLR2
TLR4
TLR5
TLR9
NOD2
AIM2
CLEC6A
CLEC4A
NLRP6
MRC1
CLEC16A
SIGNR1
RIG-1
Defb1
RELMβ
MMP7
Reg3γ
Reg3β
JAM-A
ZO-1
Jejunum
Control
IKO
Relative mRNA level
Ileum
Control
IKO
Relative mRNA level
Colon
Control
IKO
Relative mRNA level

Supplementary Fig. 7 Epithelium-associated factors gene expression in duodenum (n=6 biologically independent samples for control group and n=4 biologically independent samples for IKO group, *P* value: <0.0001, 0.0255, 0.0184), jejunum (n=6 biologically independent samples, *P* value: 0.0180, 0.0323, 0.0035, 0.0405, 0.0111, 0.0157), ileum (n=4 biologically independent samples for control group and n=3 biologically independent samples for IKO group, *P* value: 0.0086, 0.0062, 0.0301, 0.0048, 0.0239, 0.0006, 0.0022) and colon (n=4 biologically independent samples for control group and n=3 biologically independent samples for IKO group, *P* value: 0.0093, 0.0003, 0.0003) tissues in the AMPKα1$^{fl/fl}$ (Control) and AMPKα1-IKO mice fed chow diet. The boxplot elements are defined as following: center line, median; box limits, upper and lower quartiles; whiskers, 1.5 × interquartile range. *$P<0.05$, **$P<0.01$, ***$P<0.001$ and ****$P<0.0001$ by two-tailed Student's t-tests. Source data are provided as a Source Data file.

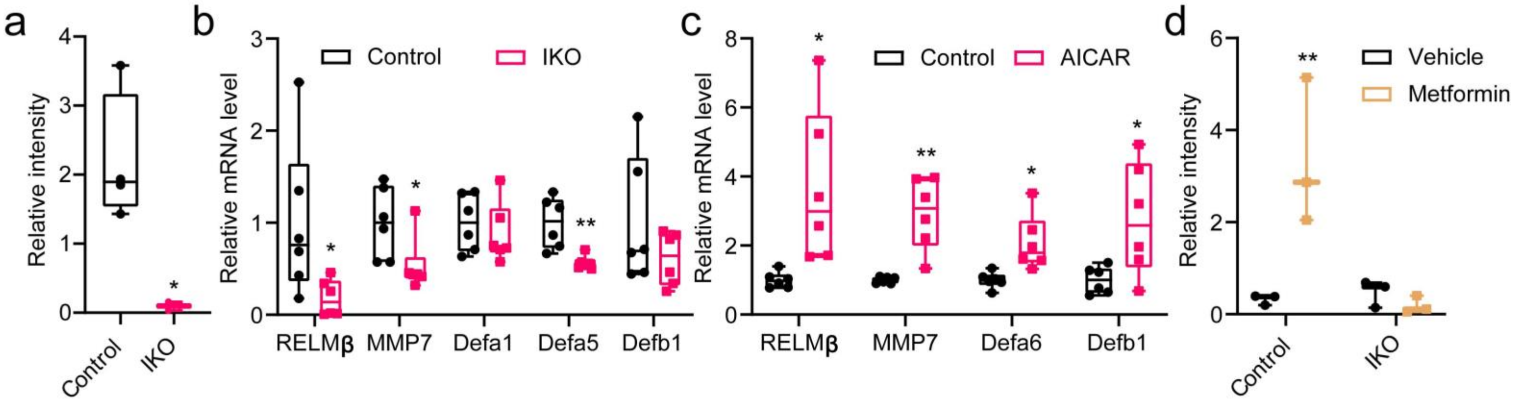

Supplementary Fig. 8 Intestinal AMPK regulates the expression of AMPs. (a) The quantified densities of the Western blot bands in Figure 3b (n=4 biologically independent samples for control group and n=3 biologically independent samples for IKO group), *P* value: 0.0132. (b) Relative mRNA levels of AMPs in the jejunum of AMPKα1$^{fl/fl}$ (Control) and AMPKα1-IKO mice fed chow diet (n=6 biologically independent samples), *P* value: 0.0438, 0.0405, 0.0040. (c) Relative mRNA levels of AMPs in HT-29 cells treated with 500 μM of AICAR (n=6 biologically independent samples), *P* value: 0.0161, 0.0011, 0.0108, 0.0275. (d) The quantified densities of the Western blot bands in Figure 3h (n=3 biologically independent samples), *P* value: 0.0040. The boxplot elements are defined as following: center line, median; box limits, upper and lower quartiles; whiskers, 1.5 × interquartile range. *$P < 0.05$, **$P < 0.01$ by two-tailed Student's t-tests (for a-c) or two-way ANOVA with Tukey's post-hoc tests for d. Source data are provided as a Source Data file.

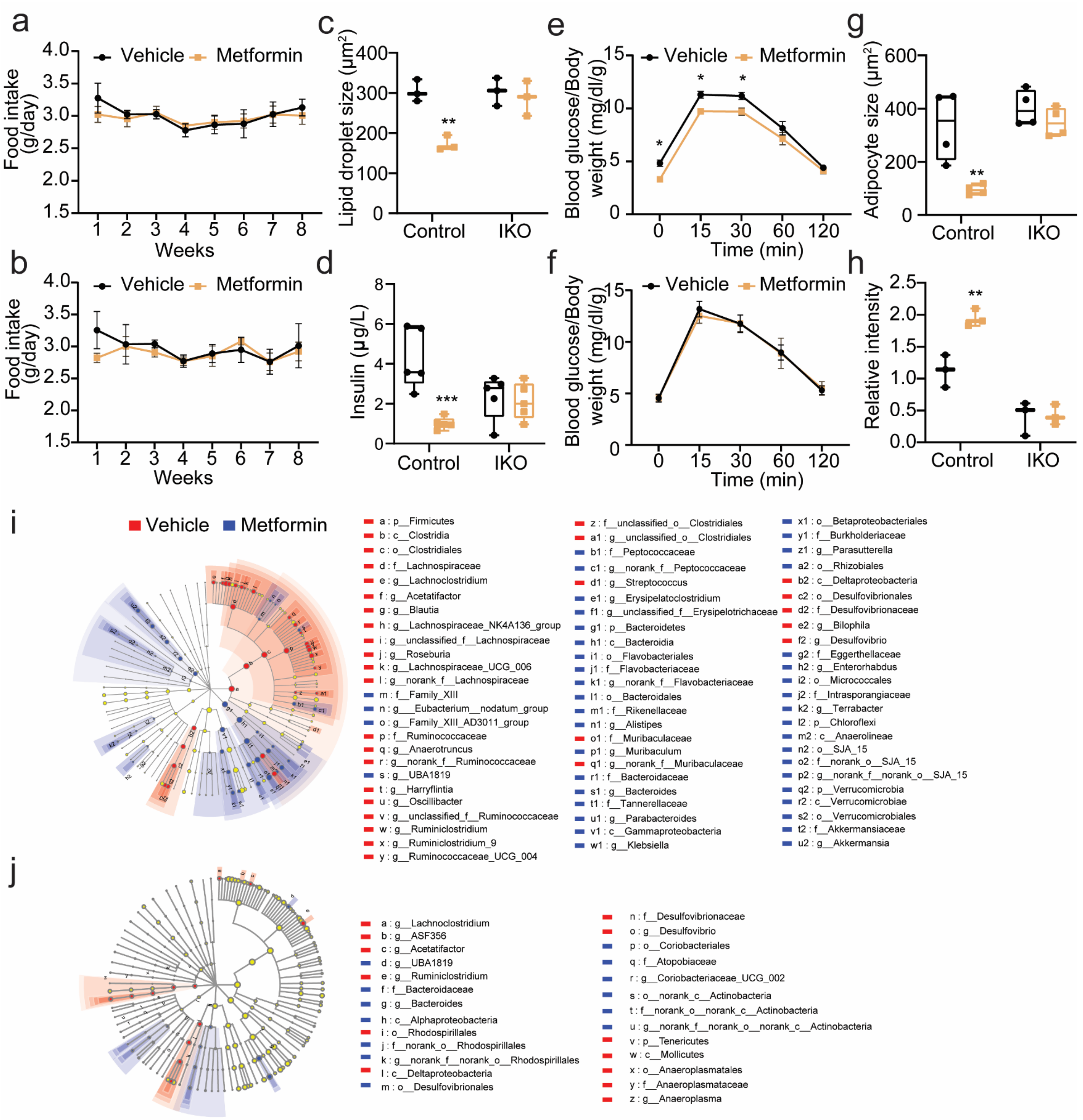
a
Vehicle
Metformin
Food intake (g/day)
4.0
3.5
3.0
2.5
2.0
1.5
1 2 3 4 5 6 7 8
Weeks
b
Vehicle
Metformin
Food intake (g/day)
4.0
3.5
3.0
2.5
2.0
1.5
1 2 3 4 5 6 7 8
Weeks
c
Lipid droplet size (μm²)
400
300
200
100
0
**
Control
IKO
d
Insulin (μg/L)
8
6
4
2
0
***
Control
IKO
e
Vehicle
Metformin
Blood glucose/Body weight (mg/dl/g)
15
10
5
0
*
0 15 30 60 120
Time (min)
f
Vehicle
Metformin
Blood glucose/Body weight (mg/dl/g)
15
10
5
0
0 15 30 60 120
Time (min)
g
Adipocyte size (μm²)
600
400
200
0
**
Control
IKO
h
Relative intensity
2.5
2.0
1.5
1.0
0.5
0.0
**
Control
IKO
i
Vehicle
Metformin
a : p__Firmicutes
b : c__Clostridia
c : o__Clostridiales
d : f__Lachnospiraceae
e : g__Lachnoclostridium
f : g__Acetatifactor
g : g__Blautia
h : g__Lachnospiraceae_NK4A136_group
i : g__unclassified_f__Lachnospiraceae
j : g__Roseburia
k : g__Lachnospiraceae_UCG_006
l : g__norank_f__Lachnospiraceae
m : f__Family_XIII
n : g__Eubacterium__nodatum_group
o : g__Family_XIII_AD3011_group
p : f__Ruminococcaceae
q : g__Anaerotruncus
r : g__norank_f__Ruminococcaceae
s : g__UBA1819
t : g__Harryflintia
u : g__Oscillibacter
v : g__unclassified_f__Ruminococcaceae
w : g__Ruminiclostridium
x : g__Ruminiclostridium_9
y : g__Ruminococcaceae_UCG_004
z : f__unclassified_o__Clostridiales
a1 : g__unclassified_o__Clostridiales
b1 : f__Peptococcaceae
c1 : g__norank_f__Peptococcaceae
d1 : g__Streptococcus
e1 : g__Erysipelatoclostridium
f1 : g__unclassified_f__Erysipelotrichaceae
g1 : p__Bacteroidetes
h1 : c__Bacteroidia
i1 : o__Flavobacteriales
j1 : f__Flavobacteriaceae
k1 : g__norank_f__Flavobacteriaceae
l1 : o__Bacteroidales
m1 : f__Rikenellaceae
n1 : g__Alistipes
o1 : f__Muribaculaceae
p1 : g__Muribaculum
q1 : g__norank_f__Muribaculaceae
r1 : f__Bacteroidaceae
s1 : g__Bacteroides
t1 : f__Tannerellaceae
u1 : g__Parabacteroides
v1 : c__Gammaproteobacteria
w1 : g__Klebsiella
x1 : o__Betaproteobacteriales
y1 : f__Burkholderiaceae
z1 : g__Parasutterella
a2 : o__Rhizobiales
b2 : c__Deltaproteobacteria
c2 : o__Desulfovibrionales
d2 : f__Desulfovibrionaceae
e2 : g__Bilophila
f2 : g__Desulfovibrio
g2 : f__Eggerthellaceae
h2 : g__Enterorhabdus
i2 : o__Micrococcales
j2 : f__Intrasporangiaceae
k2 : g__Terrabacter
l2 : p__Chloroflexi
m2 : c__Anaerolineae
n2 : o__SJA_15
o2 : f__norank_o__SJA_15
p2 : g__norank_f__norank_o__SJA_15
q2 : p__Verrucomicrobia
r2 : c__Verrucomicrobiae
s2 : o__Verrucomicrobiales
t2 : f__Akkermansiaceae
u2 : g__Akkermansia
j
a : g__Lachnoclostridium
b : g__ASF356
c : g__Acetatifactor
d : g__UBA1819
e : g__Ruminiclostridium
f : f__Bacteroidaceae
g : g__Bacteroides
h : c__Alphaproteobacteria
i : o__Rhodospirillales
j : f__norank_o__Rhodospirillales
k : g__norank_f__norank_o__Rhodospirillales
l : c__Deltaproteobacteria
m : o__Desulfovibrionales
n : f__Desulfovibrionaceae
o : g__Desulfovibrio
p : o__Coriobacteriales
q : f__Atopobiaceae
r : g__Coriobacteriaceae_UCG_002
s : o__norank_c__Actinobacteria
t : f__norank_o__norank_c__Actinobacteria
u : g__norank_f__norank_o__norank_c__Actinobacteria
v : p__Tenericutes
w : c__Mollicutes
x : o__Anaeroplasmatales
y : f__Anaeroplasmataceae
z : g__Anaeroplasma

Supplementary Fig. 9 Intestinal AMPK is required for the metabolic benefits of metformin. DIO AMPKα1$^{fl/fl}$ and AMPKα1-IKO mice were orally garaged with metformin (100 mg/kg) once daily for 8 weeks. (a-b) Daily food intake of AMPKα1$^{fl/fl}$ mice (a) and AMPKα1-IKO mice (b) (n=5 biologically independent samples). (c) Lipid droplet size analysis in Figure 4c and 4h (n=3 biologically independent samples), *P* value: 0.0023. (d) Fasting serum levels of insulin in AMPKα1$^{fl/fl}$ mice and AMPKα1-IKO mice (n=5 biologically independent samples), *P* value: 0.0004. (e-f) Glucose tolerance test relative to the body weight at 8 weeks of metformin administration in the AMPKα1$^{fl/fl}$ (e) and IKO (f) mice (n=7 biologically independent samples), *P* value for e: 0.0248, 0.0203, 0.0301. (g) Adipocyte size analysis in Figure 4k (n=4 biologically independent samples), *P* value: 0.0020. (h) The quantified densities of the Western blot bands in Figure 4l (n=3 biologically independent samples), *P* value: 0.0031. (i-j) Cladogram analysis from the phylum to genus level in AMPKα1$^{fl/fl}$ mice (i) and AMPKα1-IKO mice (j) (n=5 biologically independent samples). The different color nodes represent the microbial groups with significant enrichment in the corresponding groups. Circles indicate phylogenetic levels from domain to genus. The diameter of each circle is proportional to the abundance of the group. The yellow nodes represent the microbial groups that have no significant difference in different groups. Values are means ± s.e.m. for a-b, e-f. The boxplot elements (for c-d, g-h) are defined as following: center line, median; box limits, upper and lower quartiles; whiskers, 1.5 × interquartile range. *P<0.5, **P <0.01 and ***P <0.01 by two-way ANOVA with Tukey's post-hoc tests. Source data are provided as a Source Data file.

Supplementary Table 1 Clinical characteristics.

Characters	Patients without obesity and diabetes	Patients with obesity and diabetes	P value
Age	51.2±2.5	51.0±3.9	0.9332
Gender, M/F	3/2	3/2	
BMI	23.62±2.44	36.42±2.35	<0.0001
HbA1c (%)	5.44±0.27	8.58±1.59	0.0046

Table 1. Clinical characteristics. Data was presented as mean value ± standard deviation. T2D, type II diabetes; BMI, Body mass index. Two tailed-Student's t-tests were used to determine the statistical significance of differences between two groups.

Supplementary Table 2. Sequences of primers for qPCR.

Name	Forward primer (5'-3')	Reverse primer (5'-3')
m_Ucp1	GTACACCAAGGAAGGACCGA	TTTATTCGTGGTCTCCCAGC
m_ADRB3	GGCCCTCTCTAGTTCCCAG	TAGCCATCAAACCTGTTGAGC
m_IRF4	TCCGACAGTGGTTGATCGAC	CCTCACGATTGTAGTCCTGCTT
m_Dio2	AATTATGCCTCGGAGAAGACCG	GGCAGTTGCCTAGTGAAAGGT
m_PGC-1α	TATGGAGTGACATAGAGTGTGCT	CCACTTCAATCCACCCAGAAAG
m_Elovl3	TTCTCACGCGGGTTAAAAATGG	GAGCAACAGATAGACGACCAC
m_Cox8	TGTGGGGATCTCAGCCATAGT	AGTGGGCTAAGACCCATCCTG
m_Prdm16	CCAAGGCAAGGGCGAAGAA	AGTCTGGTGGGATTGGAATGT
m_Aco2	ATCGAGCGGGGAAAGACATAC	TGATGGTACAGCCACCTTAGG
m_Atp5a1	TCTCCATGCCTCTAACACTCG	CCAGGTCAACAGACGTGTCAG
m_Ndufb8	TGTTGCCGGGGTCATATCCTA	AGCATCGGGTAGTCGCCATA
m_Sdhb	AATTTGCCATTTACCGATGGGA	AGCATCCAACACCATAGGTCC
m_Uqcrc2	AAAGTTGCCCCGAAGGTTAAA	GAGCATAGTTTTCCAGAGAAGCA
m_Pparα	AGAGCCCCATCTGTCCTCTC	ACTGGTAGTCTGCAAAACCAAA
m_Cidec	ATGGACTACGCCATGAAGTCT	CGGTGCTAACACGACAGGG
m_Cpt2	CAGCACAGCATCGTACCCA	TCCCAATGCCGTTCTCAAAAT
m_Acox1	TAACTTCCTCACTCGAAGCCA	AGTTCCATGACCCATCTCTGTC
m_Mcad	AGGGTTTAGTTTTGAGTTGACGG	CCCCGCTTTTGTCATATTCCG
m_ATGL	GGATGGCGGCATTTCAGACA	CAAAGGGTTGGGTTGGTTCAG
m_Scd1	TTCTTGCGATACACTCTGGTGC	CGGGATTGAATGTTCTTGTCGT
m_36B4	AGATTCGGGATATGCTGTTGGC	TCGGGTCCTAGACCAGTGTTC
m_Reg3γ	ATGCTTCCCCGTATAACCATCA	GGCCATATCTGCATCATACCAG
m_Reg3β	ACTCCCTGAAGAATATACCCTCC	CGCTATTGAGCACAGATACGAG
m_RELMβ	AAGCCTACACTGTGTTTCCTTTT	GCTTCCTTGATCCTTTGATCCAC
m_Defb1	AGGTGTTGGCATTCTCACAAG	GCTTATCTGGTTTACAGGTTCCC
m_MMP7	CTGCCACTGTCCCAGGAAG	GGGAGAGTTTTCCAGTCATGG
m_Defa1	TCAAGAGGCTGCAAAGGAAGAGAAC	TGGTCTCCATGTTCAGCGACAGC
m_Defa5	TCAAAAAAGCTGATATGCTATTG	AGCTGCAGCAGAATACGAAAG
h_Reg3a	AGCTACTCATACGTCTGGATTGG	CACCTCAGAAATGCTGTGCTT
h_RELMβ	CCGTCCTCTTGCCTCCTTC	CTTTTGACACTAGCACACGAGA
h_Defb1	ATGAGAACTTCCTACCTTCTGCT	TCTGTAACAGGTGCCTTGAATTT
h_Defa6	CTGAGCCACTCCAAGCTGAG	GTTGAGCCCAAAGCTCTAAGAC
h_MMP7	GAGTGAGCTACAGTGGGAACA	CTATGACGCGGGAGTTTAACAT
h_36B4	AACATGCTCAACATCTCCCC	CCGACTCCTCCGACTCTTC
