# Supplementary material for: Intestinal AMPK modulation of microbiota mediates crosstalk with brown fat to control thermogenesis
Source: Nat Commun. 2022 Mar 3;13:1135. doi: 10.1038/s41467-022-28743-5 (PMC8894485; doi:10.1038/s41467-022-28743-5)
Supplement: Supplementary file 3 — Reporting Summary [file 41467_2022_28743_MOESM3_ESM.pdf]

nature research

Corresponding author(s): Wendong Huang, Li Yang

Last updated by author(s): Dec. 3, 2021

Reporting Summary

Nature Research wishes to improve the reproducibility of the work that we publish. This form provides structure for consistency and transparency in reporting. For further information on Nature Research policies, see our Editorial Policies and the Editorial Policy Checklist.

Statistics

For all statistical analyses, confirm that the following items are present in the figure legend, table legend, main text, or Methods section.

n/a	Confirmed	
☐	☒	The exact sample size (*n*) for each experimental group/condition, given as a discrete number and unit of measurement
☐	☒	A statement on whether measurements were taken from distinct samples or whether the same sample was measured repeatedly
☐	☒	The statistical test(s) used AND whether they are one- or two-sided<br>*Only common tests should be described solely by name; describe more complex techniques in the Methods section.*
☐	☒	A description of all covariates tested
☐	☒	A description of any assumptions or corrections, such as tests of normality and adjustment for multiple comparisons
☐	☒	A full description of the statistical parameters including central tendency (e.g. means) or other basic estimates (e.g. regression coefficient) AND variation (e.g. standard deviation) or associated estimates of uncertainty (e.g. confidence intervals)
☐	☒	For null hypothesis testing, the test statistic (e.g. *F*, *t*, *r*) with confidence intervals, effect sizes, degrees of freedom and *P* value noted<br>*Give P values as exact values whenever suitable.*
☒	☐	For Bayesian analysis, information on the choice of priors and Markov chain Monte Carlo settings
☐	☒	For hierarchical and complex designs, identification of the appropriate level for tests and full reporting of outcomes
☐	☒	Estimates of effect sizes (e.g. Cohen's *d*, Pearson's *r*), indicating how they were calculated

Our web collection on statistics for biologists contains articles on many of the points above.

Software and code

Policy information about availability of computer code

Data collection	UHPLC-MS raw data files were converted into mzXML format using the “msconvert” program from Pro-teoWizard and then analyzed by the XCMS 67 and CAMERA toolbox 68 with R statistical software. For the Western blot analysis, protein levels were quantified by densitometry using Image J software.
Data analysis	All the software and codes used in current study are either commercial or have been previously published. Detailed information are provided in the text. No custom code has been developed and applied in current study.

For manuscripts utilizing custom algorithms or software that are central to the research but not yet described in published literature, software must be made available to editors and reviewers. We strongly encourage code deposition in a community repository (e.g. GitHub). See the Nature Research guidelines for submitting code & software for further information.

Data

Policy information about availability of data

All manuscripts must include a data availability statement. This statement should provide the following information, where applicable:

- Accession codes, unique identifiers, or web links for publicly available datasets
- A list of figures that have associated raw data
- A description of any restrictions on data availability

All of the source data are provided with this paper. The source data underlying Figs. 1–4 and Supplementary Figs. 1–9 are provided as a Source Data file. The 16S rRNA sequencing data that support the findings of this study are available through the NCBI accession code PRJNA753804 (https://www.ncbi.nlm.nih.gov/sra/PRJNA753804). The metabolomics data generated in this study are available through Metabolomics Workbench accession code ST001959 (https://www.metabolomicsworkbench.org/data/DRCCMetadata.php?Mode=Study&StudyID=ST001959).

Field-specific reporting

Please select the one below that is the best fit for your research. If you are not sure, read the appropriate sections before making your selection.

☒ Life sciences ☐ Behavioural & social sciences ☐ Ecological, evolutionary & environmental sciences

For a reference copy of the document with all sections, see nature.com/documents/nr-reporting-summary-flat.pdf

Life sciences study design

All studies must disclose on these points even when the disclosure is negative.

Sample size	Sample sizes were not predetermined based on statistical methods, but were chosen according to the standards of the field (at least three independent biological replicates for each condition), which generated a sufficient number of single-molecule trajectories and gave sufficient statistics for the effect sizes of interest.
Data exclusions	No data were excluded from the analyses.
Replication	To verify the reproducibility of our findings, experiments were performed using at least three biological replicates with similar results.
Randomization	For all the experiments, samples were randomized into groups.
Blinding	For all the experiments, investigators were blinded to group allocation during data collection.

Reporting for specific materials, systems and methods

We require information from authors about some types of materials, experimental systems and methods used in many studies. Here, indicate whether each material, system or method listed is relevant to your study. If you are not sure if a list item applies to your research, read the appropriate section before selecting a response.

Materials & experimental systems

n/a	Involved in the study	
☐	☒	Antibodies
☐	☒	Eukaryotic cell lines
☒	☐	Palaeontology and archaeology
☐	☒	Animals and other organisms
☐	☒	Human research participants
☒	☐	Clinical data
☒	☐	Dual use research of concern

Methods

n/a	Involved in the study	
☒	☐	ChIP-seq
☒	☐	Flow cytometry
☒	☐	MRI-based neuroimaging

Antibodies

Antibodies used	anti-pAmpk-α Thr172 (Cell Signaling, #2535s), anti-Ampk-α1 (R&D, #AF3197), anti-Ampk-α (Cell Signaling, #2532), anti-Ampk-α2 (abcam, ab3760), anti-UCP1 (abcam, ab10983), anti-HSP90 (Cell Signaling, #4874), anti-Bcl2 (abcam, ab196495), anti-cleaved Caspase 3 (Cell Signaling, #9661), anti-GAPDH (Cell Signaling, #2118), anti-Reg3γ (abcam, ab198216), anti-Reg3α (abcam, ab202057) and mouse anti-rabbit IgG-HRP (Cell Signaling, #7074S)
Validation	All the used antibodies are commercially available and have been validated by the following manufacturers BD Biosciences, Cell Signaling, and abcam. Validation reports can be found on their websites using the catalogue number indicated above.

Eukaryotic cell lines

Policy information about cell lines

Cell line source(s)	HT29 cell lines were purchased from ATCC, HIB1B cell lines were kindly provided to us by Dr. Ke Ma at Beckman research Institute, Duarte, CA, USA. The original source of HIB1B cell line was from Spiegleman lab that generated this line (PMID: 1323843).
Authentication	None of the cell lines used were authenticated.
Mycoplasma contamination	The cell lines were not tested for mycoplasma contamination

Commonly misidentified lines (See ICLAC register)	None of the misidentified lines were used.

Animals and other organisms

Policy information about studies involving animals; ARRIVE guidelines recommended for reporting animal research

Laboratory animals	Wild-type (WT) C57BL/6J, C57BL/6-AMPKa1flox/flox mice (#014141), and B6. Cg-Tg (Vil-cre) 1000Gum/J (#021504) were purchased from Jackson Laboratory (Bar Harbor, ME). AMPK α1-IKO mice were gener-ated by cross-breeding C57BL/6-AMPKa1flox/flox mice with B6. Cg-Tg (Vil-cre) 1000Gum/J. All animal procedures were approved by the City of Hope Institutional Animal Care and Use Committee (IACUC No. 14031) and conducted in accordance with the National Institutes of Health Guidelines for the Care and Use of Laboratory Animals. 8-12-week-old male mice were used in the animal experiments. Mice were housed in a temperature (22-23°C) and light-controlled vivarium with free access to water and normal chow diet (17% kcal fat; Diet 8640, Harlan Teklad, Madison, WI) or high-fat diet (HFD, 60% kcal fat; D12492, Research Diets, New Brunswick, NJ).
Wild animals	The study did not involve wild animals.
Field-collected samples	The study did not involve samples collected from the field.
Ethics oversight	Experiments were approved by the Institutional Animal Care and Use Committees of City of Hope, (Approved protocol: IACUC 14031)

Note that full information on the approval of the study protocol must also be provided in the manuscript.

Human research participants

Policy information about studies involving human research participants

Population characteristics	46 to 58 years old healthy subjects and patients with diabetes and obesity were included in the study. Type 2 diabetes was diagnosed according to the American Diabetes Association diagnostic criteria (American Diabetes Association 2012). The information of healthy subjects and diabetic patients are shown in the Supplementary Table S1. All clinical duodenal mucosa specimens were collected from patients at the Southern California Islet Cell Resource Center at City of Hope.
Recruitment	All the patients were recruited at the Southern California Islet Cell Resource Center at City of Hope. In brief, subjects were divided into an obese with diabetes group (n=5) and a non-obese, non-diabetic group (n=5). Obesity was defined as a body mass index of 30 or greater, and T2D was defined according to the stringent HbA1c guidelines established by the American Diabetes Association (T2D: HbA1c >6.5%, approximately equivalent to 7.8 mM blood glucose) (Supplementary Table 1).
Ethics oversight	All participants provided written informed consent, and ethical approval for this study was granted by the Institutional Review Board of City of Hope (IRB No. 01046).

Note that full information on the approval of the study protocol must also be provided in the manuscript.
